# Color Stability of 3D-Printed Dental Resins Following Different Surface Treatments

**DOI:** 10.3390/polym18080901

**Published:** 2026-04-08

**Authors:** Agnieszka Nowakowska-Toporowska, Zbigniew Raszewski, Adam Nowicki, Joanna Weżgowiec, Julita Kulbacka, Edward Kijak

**Affiliations:** 1 Department of Prosthodontics, Wroclaw Medical University, Krakowska 26, 50-425 Wroclaw, Poland; edward.kijak@umw.edu.pl; 2 SpofaDental, Markova 238, 506-01 Jicin, Czech Republic; 3 Diamante Dental Clinics, ul. Sportowa 48A/C, 59-300 Lubin, Poland; 4 Department of Experimental Dentistry, Wroclaw Medical University, 50-425 Wroclaw, Poland; joanna.wezgowiec@umw.edu.pl; 5 Department of Molecular and Cellular Biology, Faculty of Pharmacy, Wroclaw Medical University, Borowska 211A, 50-556 Wroclaw, Poland; 6 Department of Immunology and Bioelectrochemistry, State Research Institute Centre for Innovative Medicine, LT-08406 Vilnius, Lithuania

**Keywords:** color, 3D printing, post-processing

## Abstract

Introduction: Recent advancements in technologies, such as 3D printing, have been adopted in prosthodontics to streamline clinical procedures and provide high-quality prosthetic devices to patients within a reduced timeframe. Aim of the study: This study primarily aimed to determine the color change levels of 3D-printed dental resins for temporary and long-term intraoral applications. We also evaluated the effectiveness of post-processing procedures such as polishing or glazing on color stability. Materials and methods: Three types of dental resins were tested in distilled water, coffee, and wine environments for 2, 7, 30, and 60 days. A spectrophotometric analysis was conducted, and the Ciede2000 formula was used to determine the DE. Results: The material type, conditioning method, and storage time significantly affected the color changes of the tested materials. The post-processing technique had the most remarkable impact on color stability over time. Conclusions: Glazing of the 3D-printed material surface appears to be the most effective approach to prolong its clinical applicability by maintaining color stability.

## 1. Introduction

Recent advances in the field of prosthodontics are aimed at enhancing clinical procedures and providing high-quality, precise, and homogenic prosthetic devices for patients in a timely manner. Ongoing research focuses on the development of superior materials and refinement of processing techniques to make them more efficient, precise, predictable, and cost-effective. Computer-aided design/computer-aided manufacturing (CAD/CAM) technologies, such as subtractive manufacturing (SM) and additive manufacturing (AM), are based on three-dimensional printing and are widely used in dentistry [1,2,3].

Subtractive-manufactured dental appliances exhibit superior mechanical properties because of the use of pre-polymerized discs fabricated under strict temperature and pressure control. This yields a highly cross-linked polymer with minimal residual monomer and eliminates polymerization shrinkage [4]. Subtractive techniques have several disadvantages, including substantial material loss and a high wear rate of the milling burrs [5]. Additionally, this method has limitations, such as the restricted motion range of the cutting device and the size of the cutting bur, which limit the availability of millable shapes [6]. It may also induce microscopic cracks that weaken the restoration over time. Moreover, marginal defects in the product can occur due to material chipping in sharp areas of the preparation [1].

Additive manufacturing is used for preparing models, digital wax-ups, surgical guides, gingival masks, splints, and temporary and permanent prosthetic appliances such as single-tooth restorations, bridges, and partial and complete dentures. Compared to subtractive milling, additive manufacturing is more advantageous, as it saves material [7,8,9]. For oral appliances, the 3D-printing process designed for generating medical resins requires high-quality and hygienic laboratory conditions. A separate, dedicated printing device should be installed in the dental laboratory to produce intraoral appliances. The most widely used techniques for printing light-cured resins are mask stereolithographic printing (SLA) and digital light processing (DLP), which offer high accuracy and fine surface finish [10,11,12].

Three-dimensional additive techniques produce objects through layer-by-layer polymerization of the liquid photopolymer in a chamber by using an ultraviolet laser with a strong light source or an LCD panel [13]. Despite the various technologies used in 3D printers, the main components of 3D printing resins are acrylate and methacrylate resins.

However, this polymerization method has some limitations, such as the presence of more residual monomers in the polymeric material, reduced double bond conversion, and decreased interlayer bonding [14]. Many studies have reported that conventional or milled CAD/CAM blocks show superior performance compared to 3D-printed resins in terms of mechanical properties and color stability [13,14,15]. Therefore, these resins are mainly used for temporary or long-term used temporary restorations [16].

The dental materials used in prosthodontics should provide long-term stability. This requires favorable mechanical properties such as flexural strength, hardness, impact strength, resistance to bacterial and fungal contamination, smoothness and resistance to staining, adequate esthetics, and a wide range of available color shades. Surface roughness of the dental materials is a critical issue for long-term color stability and restricting gingival and plaque indices [17,18].

Post-processing methods were introduced to reduce surface roughness and increase the biocompatibility of 3D-printed resins [19,20]. These include polishing and glazing with light-cured, low-viscosity materials. Polishing and glazing provide initial advantages in lowering the material roughness and preventing discoloration; however, the effectiveness of these procedures over time are unknown.

Color change primarily indicates material aging. Color change can be precisely measured by spectrophotometry. Compared to the CIELab formula, the Ciede2000 color difference formula can more precisely and better reflect the color differences perceived by the human eye [21]. The color change perceptible to the human eye is 3–3.5 on the Cielab2000 scale. In dentistry, the perceptibility threshold is 1.72, and the acceptable color change level cannot exceed 4.08 [22]. Color alteration reduces the acceptability of the product by the patient, leading to more frequent replacement of prosthetic devices.

In this context, the present study aimed to determine the color change levels of 3D-printed dental resins for temporary and long-term intraoral use. Additionally, the study evaluated whether additional post-processing procedures such as polishing or glazing could be an effective method to provide higher color stability over time. The null hypothesis assumed for this study was that the material type, conditioning method, and storage time do not affect color change of the tested materials in distilled water and two colorant solutions (coffee and red wine).

## 2. Materials and Methods

### 2.1. Specimen Preparation

Disk-shaped specimens (10 mm diameter and 1 mm height) were designed for this study. The discs, prepared from two composites (CB and 3D) for long-term prosthetic restorations, were printed using the NextDent 5100 printer (NextDent BV., Soesterberg, The Netherlands) under digital light processing Table 1. The specimens of composite for temporary restorations (Temp specimens) were printed with the Phrozen shuffle 2019 (Phrozen Tech Co., Hisnchu, Taiwan) LCD resin 3D printer through mask stereolithography. The printing orientation was 0°, and the layer thickness was 50 µm for all tested materials. All discs were then placed in an ultrasonic cleaner with isopropyl alcohol for 1 min and subsequently with pure ethanol for 2 min. The specimens were then irradiated for the complete conversion of unreacted monomers in the curing chamber for 30 min (CB and 3D) and for 12 min (Temp) according to the manufacturer’s instructions.

The printing supports were removed mechanically. The discs were assigned to 3 groups. The first group was raw (non-polished) material. The second group underwent a manual polishing process for 15 min using prosthetic rubbers with a decreasing abrasion gradient. The third group was glazed with GC Optiglaze (GC Corporation, Tokyo, Japan) and cured with a UV lamp (wavelength < 430 nm) for 40 s. The glaze was applied one time in a thin and uniform layer using a brush by a single experienced operator. GC Optiglaze is a light-curing glaze coating used in dentistry to provide smoothness, gloss, and stain resistance to composite materials and 3D-printed restorations [23].

A total of 45 specimens were prepared from each of the 3 tested materials. The specimens were subdivided into 3 groups based on the applied surface treatment: non-polished, polished, and glazed. The specimens were again divided into 3 subgroups depending on storage environment Table 2. The specimens were stored in separate containers filled with distilled water, coffee, or red wine at room temperature (23 °C) in darkness.

The coffee solution was prepared by adding three teaspoons of 10.0 g ground coffee (Jacobs Kronung, Jacobs Douwe Egberts, Warsaw, Poland) to 250 mL boiling water; the resulting mixture was brewed for 10 min to prepare the test solution. This solution was then filtered through a coffee filter to remove residues and cooled to room temperature. The wine used in this experiment was a semi-sweet red wine (Kazbek Peak Saperavi, Tbilisi, Georgia). The alcohol and sugar contents were 12% and 25 g/L, respectively. The measured pH value of the tested wine was 3.6 (previously calibrated with standard solutions of pH 3 and 5 (pH Meter NineFocus NF2000 (Mettler Toledo, Prague, Czech Republic); pH sensor InLab Expert Pro-DES electrode (Mettler Toledo, Prague, Czech Republic))) [24].

The volume of each solution was 25 mL. The solutions were changed weekly during the entire experimental duration.

### 2.2. Color Measurement

The initial color of the specimens was established as DE0. Subsequently the color was assessed after 2, 7, 30, and 60 days of storage in two colorant solutions (coffee and red wine) and distilled water. Before each measurement, the specimens were removed from the storage container, washed with tap water, and dried with a paper towel.

Color measurements were conducted using the X-Rite S 23 spectrophotometer (X Rite Inc., Grand Rapids, MI, USA). Spectrophotometry is an objective method of assessing color change, and it measures the amount of light reflected by the specimen. This facilitates the determination of DE, which is defined as the change in the position of a point in the three-dimensional CIE L*a*b* space Before each series of measurements, the spectrophotometer was calibrated according to the manufacturer’s instructions by using its white reference tile that corresponds to the L (lightness) parameter. Prior to the measurement, the samples were mounted in silicone matrix (Elite HD, Zhermack, Badia Polesine, Italy) to allow the same measurement location for each specimen. The measurements were then performed under temperature-controlled laboratory conditions at 23 °C and 50% humidity. The L value of 100 is achieved by the ideal white, and the L value of 0 by the ideal black. Values of a and b parameters describe the color scale: positive and negative a values represent red and green, respectively, while positive and negative b values denote yellow and blue, respectively. A measurement cleft of 4 mm was maintained throughout the entire study. Color measurements were performed three times for each specimen, and the average value was recorded. L* is the lightness, whereas “a*” (green~magenta) and “b*” (blue~yellow) are the chromatic axes. L*, a*, and b* values measured at each time point were applied to tones induced by the colorants. The ∆E00 values were calculated using the following equation:

$${∆E}_{00}=\sqrt{{\left(\frac{{∆L}^{\prime }}{K_{L}S_{L}}\right)}^{2}}+{\left(\frac{{∆C}^{\prime }}{K_{C}S_{C}}\right)}^{2}+{\left(\frac{{∆H}^{\prime }}{K_{H}S_{H}}\right)}^{2}+R_{T\;}\left(\frac{{∆C}^{\prime }}{K_{C}S_{C}}\right)\left(\frac{{∆H}^{\prime }}{K_{H}S_{H}}\right)$$

*S_L_*, *S_C_*, and *S_H_* are the functions to calibrate the absence of visual uniformity of the CIE Lab formula on the direction of lightness (*L*), chroma (*C*), and hue (*H*). *K_L_*, *K_C_*, and *K_H_* are the correction parameters of the environment. L*, a*, and b* values were measured on a white background, and the parametric values of *K_L_ K_C_*, and *K_H_* were set to 1 [25].

### 2.3. Statistical Analysis

To examine the null hypothesis assumed for this study, a two-way repeated-measures ANOVA was conducted separately for each tested environment. To validate the applicability of this method, Mauchly’s test of sphericity was performed for the variance of differences between the measurements. If the assumption of sphericity was not met, the Greenhouse–Geisser correction was applied to the results of two-way repeated-measures ANOVA. Post hoc Tukey tests were performed to determine differences between pairs. Differences with *p* < 0.05 were considered statistically significant.

## 3. Results

### 3.1. Distilled Water

For the between-subject effects, the material type showed a significant effect (F(2, 40) = 8.38; *p* < 0.001, η^2^p = 0.30 (95% CI [0.10, 0.47]), together with the conditioning method (F(2, 40) = 37.38; *p* < 0.001, η^2^p = 0.65 (95% CI [0.48, 0.76]). This finding indicates that the mean color change exhibited a significant difference depending on the material type and the applied surface treatment. The analysis also revealed a very strong main effect of exposure time (F(2.24, 89.52) = 306.52; *p* < 0.001, η^2^p = 0.88 (95% CI [0.82, 0.92]), demonstrating clear differences in color change across the measurement days. Significant interactions were additionally observed for exposure time × material (F(4.48, 89.52) = 10.91; *p* < 0.001, η^2^p = 0.35 (95% CI [0.18, 0.48]) and exposure time × conditioning (F(4.48, 89.52) = 25.86; *p* < 0.001, η^2^p = 0.56 (95% CI [0.40, 0.67]). These findings suggest that the dynamics of color change depend on both material type and conditioning method, with exposure time exerting the strongest and most precise effect Table 3.

### 3.2. Material-Dependent Color Change in Distilled Water

**Figure 1:**
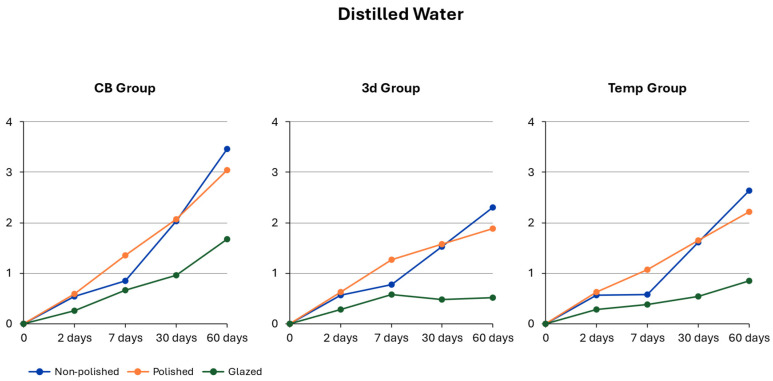
Time-dependent color change in distilled water.

The results of post hoc Tukey tests indicated no significant difference in color change levels between the tested materials after the 2nd and 7th days of the experiment. After day 30, color alteration for CB material was significantly higher than for 3D material; furthermore, after day 60, CB material showed significantly higher color alteration than both 3D and Temp materials Figure 1.

### 3.3. Conditioning-Dependent Color Change in Distilled Water

The results of post hoc Tukey tests revealed significantly lower discoloration for glazed specimens of all tested materials after days 2, 30, and 60 of the experiment in comparison to polished and non-polished specimens.

On day 2 of the experiment, glazed specimens of all tested materials had significantly lower color change values (CB DE = 0.26 ± 0.02; Temp DE = 0.28 ± 0.02; 3D DE = 0.28 ± 0.02) compared to non-polished and polished specimens.

On day 7 of the experiment, glazed specimens continued to have significantly lower discoloration values (CB DE = 0.66 ± 0.15; Temp DE = 0.38 ± 0.15; 3D DE = 0.58 ± 0.15) compared to polished specimens.

On day 30 of the experiment, the discoloration values of glazed specimens were lower than those of both non-polished and polished specimens (CB DE = 0.96 ± 0.12; Temp DE = 0.54 ± 0.12; 3D DE = 0.47 ± 0.12). This trend continued on the 60th day of the experiment and was even more pronounced (CB DE = 1.67 ± 0.20; Temp DE = 0.85 ± 0.20; 3D DE = 0.51 ± 0.20) (Table 4).

The present study data showed that non-polished and polished specimens of CB material had the highest color change after the 60th day of storage in distilled water. In contrast, the glazed 3D specimens exhibited the lowest color change.

Although both material type and conditioning method substantially affected color change, the conditioning method appears to have considerably greater influence Figure 1.

Regarding perceptibility thresholds, the color change was not perceptible for all materials despite the conditioning method after the 2nd and 7th day of the experiment. On the 30th day of the experiment, perceptible discoloration was noted for CB in the non-polished and polished groups. On day 60, no perceptible discoloration was noted for the glazed groups of all tested materials. All materials in all environments were below the acceptance level of 4.08 throughout the entire experiment.

### 3.4. Coffee

For the between-subject effects, the material type showed a significant influence (F(2, 40) = 8.81; *p* < 0.001, η^2^p = 0.34 (95% CI [0.12, 0.50]), together with the conditioning method (F(2, 40) = 82.38; *p* < 0.001, η^2^p = 0.80 (95% CI [0.70, 0.86]). This finding indicates that the mean color change differed significantly depending on material type and the applied surface treatment. A significant main effect of exposure time was also observed (F(1.17, 46.90) = 344.47; *p* < 0.001, η^2^p = 0.90 (95% CI [0.85, 0.93]), showing clear differences in color change across the individual exposure days. Significant interaction effects were also noted for material type (F(2.34, 46.90) = 10.41; *p* < 0.001, η^2^p = 0.34 (95% CI [0.16, 0.48]) and the conditioning method (F(2.34, 46.90) = 57.29; *p* < 0.001, η^2^p = 0.74 (95% CI [0.62, 0.82]). Similarly to the results obtained in the distilled water environment, exposure time exerted the strongest and most precise influence on color change, whereas material type showed the weakest effect Table 4. Exposure time also had a higher impact on color change in coffee than in distilled water.

### 3.5. Material-Dependent Color Change in Coffee

**Figure 2:**
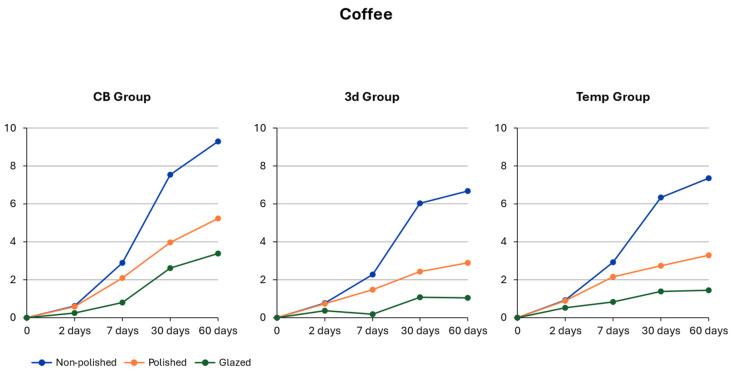
Time-dependent color change in coffee.

The results of post hoc Tukey tests indicated a significant difference in color change levels between CB material (lowest discoloration) and Temp material (highest discoloration) on day 2 of the experiment. On day 7, color change was significantly different between CB and Temp materials, and both these materials demonstrated a higher DE than 3D material. On day 30, the tested materials exhibited no significant differences in color change. Finally, on day 60, CB material showed significantly higher discoloration than both 3D and Temp materials Figure 2.

### 3.6. Conditioning-Dependent Color Change in Coffee

On day 2 of the experiment, the DE values obtained for glazed specimens of all tested materials was significantly lower (CB *DE* = 0.23 ± 0.04; Temp *DE* = 0.52 ± 0.04; 3D *DE* = 0.36 ± 0.04) than those for non-polished and polished materials. The DE of all tested groups showed significant differences after the 7th and 60th days of the experiment.

After 7 days, glazed specimens exhibited significantly lower levels of color change (CB *DE* = 0.79 ± 0.14; Temp *DE* = 0.83 ± 0.14; 3D *DE* = 0.17 ± 0.14). Simultaneously, the non-polished group achieved the highest values for the DE levels (CB *DE* = 2.88 ± 0.14; Temp *DE* = 2.93 ± 0.14; 3D *DE* = 2.26 ± 0.14).

The same observation was made after 30 days of storage. The glazed specimens had the lowest color change values (CB *DE* = 2.61 ± 0.42; Temp *DE* = 1.39 ± 0.42; 3D *DE* = 1.07 ± 0.42), while DE values for the non-polished specimens were significantly higher (CB *DE* = 7.56 ± 0.42; Temp *DE* = 6.33 ± 0.42; 3D *DE* = 6.02 ± 0.42).

After the 60th day of the experiment, DE values for the glazed specimens were significantly lower (CB *DE* = 3.38 ± 0.46; Temp *DE* = 1.44 ± 0.46; 3D *DE* = 1.04 ± 0.46); however, the non-polished group continued to show significantly higher DE values (CB *DE* = 9.30 ± 0.46; Temp *DE* = 7.36 ± 0.46; 3D *DE* = 6.96 ± 0.46).

The non-polished specimens had significantly higher DE values after 60 days of immersion in coffee, while the glazed group exhibited the lowest level of DE. Regarding the perceptibility of color change, coffee caused no perceptible discoloration of all tested groups after 2 days of the experiment. On day 7, all non-polished and polished groups of CB and Temp materials exhibited perceptible color changes. Finally, on days 30 and 60, only glazed Temp and 3D materials showed no perceptible discoloration. The acceptability threshold was exceeded after the 30th and 60th day by all non-polished groups and by the polished CB group.

### 3.7. Red Wine

For the between-subject effects, the material type exhibited a significant influence (F(2, 40) = 10.72; *p* < 0.001, η^2^p = 0.41 (95% CI [0.20, 0.55]), together with the conditioning method (F(2, 40) = 57.65; *p* < 0.001, η^2^p = 0.74 (95% CI [0.62, 0.82]). This result indicated a significant difference in mean color change depending on material type and the applied surface treatment. A significant main effect of exposure time was also noted (F(1.05, 41.94) = 208.85; *p* < 0.001, η^2^p = 0.84 (95% CI [0.76, 0.89]), demonstrating clear differences in color change across the individual exposure days. Significant interaction effects were additionally found for material type (F(2.10, 41.94) = 13.67; *p* < 0.001, η^2^p = 0.41 (95% CI [0.22, 0.55]) and conditioning F(2.10, 41.94) = 26.06; *p* < 0.001, η^2^p = 0.62 (95% CI [0.47, 0.73]). Similarly to the results obtained in the distilled water and coffee environments, exposure time exerted the strongest influence on color change, whereas material type showed the weakest effect Table 5.

### 3.8. Material-Dependent Color Change in Wine

**Figure 3:**
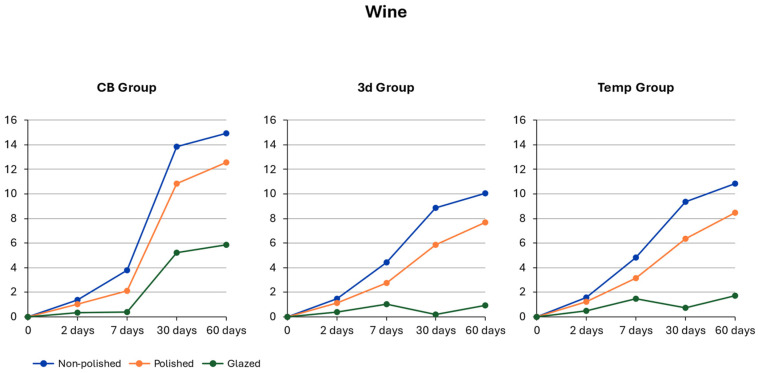
Time-dependent color change in wine.

According to the results of the post hoc Tukey tests, no significant difference was observed in color change levels between the tested materials after the 2nd day of the experiment. However, after the 7th, 30th, and 60th day of the experiment, color change for CB material was significantly higher than that for 3D and Temp materials Figure 3.

### 3.9. Conditioning-Dependent Color Change in Wine

On the 2nd day of the experiment, glazed specimens made for all tested materials exhibited the lowest color change (CB *DE* = 0.32 ± 0.07; Temp *DE* = 0.48 ± 0.07; 3D *DE* = 0.38 ± 0.07) compared to non-polished and polished specimens.

On the 7th day of the experiment, glazed specimens had significantly lower color alteration (CB *DE* = 0.38 ± 0.13; Temp *DE* = 1.45 ± 0.13; 3D *DE* = 1.03 ± 0.13), whereas non-polished specimens had the most pronounced color change (CB *DE* = 3.76 ± 0.13; Temp *DE* = 4.83 ± 0.13; 3D *DE* = 4.40 ± 0.13).

Progression was also noted in the discoloration process. On the 30th day of the experiment, glazed specimens presented the lowest discoloration (CB *DE* = 5.20 ± 0.94; Temp *DE* = 0.71 ± 0.94; 3D *DE* = 0.19 ± 0.94) in comparison to the highest values of DE for non-polished specimens (CB *DE* = 13.85 ± 0.94; Temp *DE* = 9.36 ± 0.94; 3D *DE* = 8.84 ± 0.94).

Finally, on day 60 of the experiment, glazed specimens still had significantly lower DE (CB *DE* = 5.85 ± 1.03; Temp *DE* = 1.73 ± 1.03; 3D *DE* = 0.94 ± 1.03), whereas non-polished specimens had the highest DE (CB *DE* = 14.95 ± 1.03; Temp *DE* = 10.83 ± 1.03; 3D *DE* = 10.05 ± 1.03).

Compared to glazed specimens with a significantly less pronounced discoloration process, non-polished specimens displayed the highest color change. The alteration in color was also more pronounced with the lightest initial color (CB bleach) and the least pronounced with the pink-colored 3D material. The rapid discoloration of CB material on the 30th day of the experiment is a noteworthy finding. Thus, the material type and its conditioning had a significant impact on the color change of the specimens Figure 3.

Wine caused perceptible discoloration in all non-polished and polished specimens on day 7 of the experiment. The perceptibility threshold was also exceeded in glazed specimens of CB after the 30th day of the experiment and in both CB and Temp glazed specimens after the 60th day of the experiment. The color change was above the acceptance threshold for non-polished 3D and Temp groups after the 7th day of the experiment and for all non-polished and polished groups and the glazed CB group after the 30th day of the experiment.

## 4. Discussion

The null hypothesis assumed for this study was rejected. The color change of the tested materials was significantly influenced by the material type, conditioning method, and storage time.

Conditioning method emerged as a key determinant of color stability. Glazed specimens consistently demonstrated the lowest discoloration value at every time point, whereas non-polished specimens showed the highest DE values, particularly after 30 and 60 days. This pattern highlights the protective role of a smooth, sealed surface in reducing pigment adsorption and fluid uptake. The prominent differences between the glazed and non-polished groups suggest that surface roughness and microporosity substantially accelerate staining, supporting previous findings that surface finishing is a critical factor for maintaining esthetic longevity. In the present study, the lowest color change was noted for glazed specimens, which aligns with the findings of Nam et al. [26]. Their study reported a decrease in the DE value of glazed specimens of 3D-printed resin materials in comparison to untreated and sand-glazed specimen surfaces. The positive impact of glazing may be attributed to the effect of the resin coating layer that fills the micropores and surface defects and reduces material surface porosity and microleakage [27]. The use of glazing may also reduce the sorption levels and therefore result in less discoloration [28] Glazing may remarkably enhance the quality and esthetics of 3D-printed resins. The results of our study support the findings of Almejrad et al. [29], who tested 3D-printed resins for interim restorations. The polished specimen group showed significantly higher DE than the glazed group when immersed in coffee and wine. The use of glaze provides a protective coating that can reduce discoloration caused by chromogenic beverages.

The results of our study are partially supported by the findings of Izzettinoglu et al. [30], who demonstrated that DE levels were significantly lower for only one of four tested 3D printed resins. The polished and glazed groups showed no significant differences for the other tested materials. This difference may be explained by variations in materials, production protocols, and conditioning between our study and the study by Izzettinoglu et al. 

Color change in a resin material largely depends on resin composition and filler content. Resin-based materials with lower filler volumes absorb more water, leading to hydrolytic degradation and ultimately to a greater susceptibility to staining [31]. Therefore, material composition may play a crucial role in long-term clinical success. The addition of mineral fillers may also be an important factor in determining the longevity of the dental materials.

The 3D-printed materials are more likely to become stained due to the manufacturing process. This process involves placing multiple layers on the top of one another. The possibility of incomplete polymerization at the interface of these layers, along with the presence of microporosities and residual monomers, can lead to a higher likelihood of discoloration in printed materials [32].

As suggested in previous studies, a 24 h in vitro incubation time in the colorant solution may simulate exposure to the colorants during standard food and drink consumption of approximately 30 days [33,34]. According to the estimates of numerous authors, the average consumer consumes coffee for 15 to 20 min. Among coffee drinkers, the average consumption is 3.2 cups per day, and 60 days simulate 4 years of consumption [35]. The maximum storage period of 60 days evaluated in this study is equivalent to approximately 4 years, which is appropriate for the prosthetic devices intended for long-term use.

According to the CIEDE2000 formula, the color change perceptibility threshold is 1.72, and the acceptable color change level cannot exceed 4.08 [22]. In the present study, the acceptability threshold was not exceeded by any of the tested materials in distilled water despite the use of the post-processing method. In coffee, all non-polished specimens showed discoloration above this range. The red wine environment caused the most staining in all tested materials, with perceptible discoloration observed even in glazed groups at later time points. Based on the present study data, we can conclude that all specimens underwent the discoloration process. Discoloration was the most pronounced in CB material, which may be partially explained by the lighter initial color of this material. A noteworthy observation is that both polishing and glazing reduced the discoloration process, although glazing was significantly more effective. The finding that several groups exceeded the acceptability threshold after prolonged immersion underscores the clinical importance of appropriate post-processing. Overall, these findings reinforce glazing as the most effective strategy for enhancing the long-term esthetic stability of 3D-printed provisional and long-term restorations, which aligns with previous evidence that surface smoothness and polymer cross-linking density are the key determinants of color durability.

The present study has some limitations. First, this study was only an in vitro experiment with limited tested materials, sample size, conditions, and time intervals. Second, we employed two different printing technologies (DPL and SLA) for materials used in this study. Third, the experimental design could not fully simulate the complex oral cavity environment such as impact of salivary enzymes and biofilm formation, mechanical stresses, and daily hygienic protocols performed by the patient.

Clinical applications:

Prolonged exposure to staining agents—particularly coffee and red wine—can significantly accelerate the discoloration of 3D-printed materials for both temporary and long-term use. Therefore, it is crucial to apply a smooth, sealed surface finish to 3D-printed restorations to enhance their clinical utility. Proper glazing or high-quality polishing should be considered an essential step to enhance the esthetic longevity of prosthodontic materials.

## 5. Conclusions

Within the limitations of this in vitro study, the following key conclusions can be drawn:

1. Exposure time was the dominant factor affecting color stability of all tested 3D-printed materials across distilled water, coffee, and red wine environments.
2. The conditioning method had a stronger effect on color change than the material type. Glazed specimens consistently exhibited the lowest discoloration value, while non-polished specimens showed the highest DE values in all media and at all time points.
3. Material type influenced color stability to a moderate extent. CB material demonstrated the greatest susceptibility to discoloration, particularly after long-term exposure, whereas 3D material showed the most stable color performance, particularly when glazed.
4. Staining media differed in their discoloration potential. Coffee and red wine caused more prominent color changes than distilled water, with red wine producing the highest DE values in non-polished specimens.
5. Glazing or high-quality polishing substantially improves color stability and should be considered an essential process, particularly for patients frequently consuming chromogenic beverages.

## Figures and Tables

**Table 1:** Materials, Manufacturers, Clinical Applications, and Main Components.

Material	Manufacturer	Clinical Applications	Main Components
NextDent C&B MFH (CB)	Vertex-Dental BV., The Netherlands	Temporary and permanent crowns and bridges	Microhybryd resin with inorganic fillers (Micro Filled Hybrid)
NextDent 3D Plus (3D)	Vertex-Dental BV., The Netherlands	Denture bases	Mineral fillers (SiO_2_, TiO_2_), acrylate/methacrylate monomers (including Bis-EMA, HEMA), crosslinking bismethacrylate, and the photoinitiator TPO.
Mazic D Temp (Temp)	Vericom Co., Ltd., Anyang-si, Republic of Korea	Temporary crowns and bridges	Composite resin based on methylmethacrylate(MMA) ethylene glycol dimethacrylate (EGDMA)

**Table 2:** Group divisions used in the study.

NextDent C&B MFH (45)		Nexdent 3D Plus (45)		Mazic D Temp (45)	
Non-polished (15)	Water (5)	Non-polished (15)	Water (5)	Non-polished (15)	Water (5)
	Coffee (5)		Coffee (5)		Coffee (5)
	Wine (5)		Wine (5)		Wine (5)
Polished (15)	Water (5)	Polished (15)	Water (5)	Polished (15)	Water (5)
	Coffee (5)		Coffee (5)		Coffee (5)
	Wine (5)		Wine (5)		Wine (5)
Glazed (15)	Water (5)	Glazed (15)	Water (5)	Glazed (15)	Water (5)
	Coffee (5)		Coffee (5)		Coffee (5)
	Wine (5)		Wine (5)		Wine (5)

**Table 3:** Effects of material type and conditioning method on color change in distilled water.

Effect Type	Effect	*SS*	*df*	*F*	*p*	*η* ^2^ *_p_*
Within-subject effects	Exposure time	115.30	2.24	306.52	<0.001	0.88
	Exposure time × material	8.21	4.48	10.91	<0.001	0.35
	Exposure time × conditioning	19.45	4.48	25.86	<0.001	0.56
	Residual	15.05	89.52			
Between-subject effects	Material	5.20	2	8.38	<0.001	0.30
	Conditioning	23.21	2	37.38	<0.001	0.65
	Residual	12.42	40			

*SS*—sum of squares; *df*—degrees of freedom; *F*—ANOVA test statistics; *p*—statistical significance.

**Table 4:** Effects of material type and conditioning method on color change in coffee.

Effect Type	Effect	*SS*	*Df*	*F*	*p*	*η* ^2^ *_p_*
Within-subject effects	Exposure time	699.44	1.17	344.47	<0.001	0.90
	Exposure time × material	42.26	2.34	10.41	<0.001	0.34
	Exposure time × conditioning	232.65	2.34	57.29	<0.001	0.74
	Residual	81.22	46.90			
Between-subject effects	Material	29.30	2	8.81	<0.001	0.31
	Conditioning	273.97	2	82.38	<0.001	0.80
	Residual	66.52	40			

*SS*—sum of squares; *df*—degrees of freedom; *F*—ANOVA test statistics; *p*—statistical significance.

**Table 5:** Effects of material type and conditioning method on color change in red wine environment.

Effect Type	Effect	*SS*	*Df*	*F*	*p*	*η* ^2^ *_p_*
Within-subject effects	Exposure time	2311.34	1.05	208.85	<0.001	0.84
	Exposure time × color	302.47	2.10	13.67	<0.001	0.41
	Exposure time × conditioning	576.78	2.10	26.06	<0.001	0.57
	Residual	442.69	41.94			
Between-subject effects	Material	142.45	2	10.72	<0.001	0.35
	Conditioning	766.13	2	57.65	<0.001	0.74
	Residual	265.78	40			

*SS*—sum of squares; *df*—degrees of freedom; *F*—ANOVA test statistics; *p*—statistical significance.

## Data Availability

The original contributions presented in this study are included in the article. Further inquiries can be directed to the corresponding author.
